# A Novel Methodology for Enhanced and Consistent Heterologous Expression of Unmodified Human Cytochrome P450 1B1 (CYP1B1)

**DOI:** 10.1371/journal.pone.0110473

**Published:** 2014-10-16

**Authors:** Muneeb A. Faiq, Mashook Ali, Tanuj Dada, Rima Dada, Daman Saluja

**Affiliations:** 1 Dr. Rajendra Prasad Centre for Ophthalmic Sciences, All India Institute of Medical Sciences, Ansari Nagar, New Delhi, India; 2 Medical Biotechnology Laboratory, Dr. B. R. Ambedkar Centre for Biomedical Research, University of Delhi, North Campus, Delhi, India; 3 Laboratory for Molecular Reproduction and Genetics, Department of Anatomy, All India Institute of Medical Sciences, Ansari Nagar, India; University of Michigan, United States of America

## Abstract

Cytochrome P450 1B1 (CYP1B1) is a universal cancer marker and is implicated in many other disorders. Mutations in CYP1B1 are also associated with childhood blindness due to primary congenital glaucoma (PCG). To understand the CYP1B1 mediated etiopathology of PCG and pathomechanism of various cancers, it is important to carry out its functional studies. Heterologous expression of CYP1B1 in prokaryotes is imperative because bacteria yield a higher amount of heterologous proteins in lesser time and so the expressed protein is ideal for functional studies. In such expression system there is no interference by other eukaryotic proteins. But the story is not that simple as expression of heterologous CYP1B1 poses many technical difficulties. Investigators have employed various modifications/deletions of CYP N-terminus to improve CYP1B1 expression. However, the drawback of these studies is that it changes the original protein and, as a result, invalidates functional studies. The present study examines the role of various conditions and reagents in successful and consistent expression of sufficient quantities of unmodified/native human CYP1B1 in *E. coli*. We aimed at expressing CYP1B1 in various strains of *E. coli* and in the course developed a protocol that results in high expression of unmodified protein sufficient for functional/biophysical studies. We examined CYP1B1 expression with respect to different expression vectors, bacterial strains, types of culture media, time, Isopropyl β-D-1-thiogalactopyranoside concentrations, temperatures, rotations per minute, conditioning reagents and the efficacy of a newly described technique called double colony selection. We report a protocol that is simple, easy and can be carried out in any laboratory without the requirement of a fermentor. Though employed for CYP1B1 expression, this protocol can ideally be used to express any eukaryotic membrane protein.

## Introduction

Cytochrome P450, family 1, subfamily B, polypeptide 1 (CYP1B1) is a recently identified [1] dioxin inducible aryl hydrocarbon hydroxylase with the enzyme commission number EC.1.14.14.1. It catalyses the following master reaction:
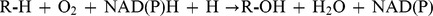



Being a member of the xenobiotics metabolizing family, CYP1B1 catalyzes the bioconversion/activation of a large number of procarcinogens but the reaction has a unique stereoselectivity and estradiol - 4 - hydroxylation is the characteristic of its catalytic actions [2]. CYP1B1 is different from both CYP1A1 and CYP1A2 in many respects. It has only 40% homology with both these genes [2]. CYP1B1 gene is located on chromosomal locus 2p21-22 [3] comprising of 3 exons and 2 introns while both CYP1A1 and CYP1A2 are located on chromosome 15 and both are arranged in 7 exons and 6 introns [2]. As confirmed by the DNA hybridization studies, CYP1B1 is the only member of CYP1B subfamily [1], [3]. Due to all the above reasons, the properties and functions of CYP1B1 cannot be predicted by the functional analysis of CYP1A1 and CYP1A2. Hence, the expression of unmodified CYP1B1 is crucial for understanding its catalytic activities, cellular functions, molecular biology and the etiopathomechanistic aspects of the diseases it is involved in.

CYP1B1 is expressed in many tissues in the body including adipose tissue, eyes, brain, colon, embryo, heart, kidneys, lungs, muscle, pancreas, testes, thymus etc. [http://www.urogene.org/pgdb/gene/107.html]. It is considered as a universal cancer marker [4]–[8] with implications in ovarian cancer [9], colorectal adenocarcinoma [10], acute lymphocytic leukemia, acute myeloid leukemia, esophageal carcinoma, lung cancers, lymphoma, rhabdomyosarcoma [6], prostate carcinoma [11] etc. In addition to this, CYP1B1 plays an important role in embryonic eye development [12]–[14] and its mutations have been implicated in primary congenital glaucoma (PCG) [15]–[17]. In our previous studies, we observed a high prevalence of CYP1B1 mutations in North Indian PCG patients and also reported 7 novel mutations [18], [19]. We have also reviewed the molecular, biochemical, diagnostic, clinical and genetic aspects of CYP1B1 involvement in PCG [20], [21].

Many investigators have reported enhanced expression of N-terminal modified CYP1B1 in *E. coli* [22], [23]. However, such a protein may not be a pertinent representative for valid functional studies for obvious reasons. Change in any aminoacid in the native protein sequence may produce numerous (predictable/unpredictable) changes in the functional integrity of the translation product and may, as a consequence, render the functional studies invalid. Previous studies have also shown that N-terminal mutations are associated with many diseases, suggesting that this region is critical for the normal function of CYP1B1 [18], . A few examples of such already reported N-terminal mutations involved in pathogenesis of PCG are L24R, P54L, W57C, I60M, G61E, L77P and Y81N [24]. Hence, sufficient amount of the purified full length protein is required for carrying out the functional studies. Membrane proteins are difficult to express in prokaryotic expression systems for many reasons sufficiently elucidated by Orawan et al. (2012) [25] and many others [26]–[28]. Cytochrome P450s are particularly difficult to express in procaryotic expression systems as there is often the possibility of formation of self hybridization derived secondary structures in mRNA transcripts [29]. This issue becomes more significant as protein purification also leads to considerable protein loss. Hence, a reliable method/protocol to consistently express membranous proteins is, therefore, extremely important. Since unmodified CYP1B1 expression has not been reported so far to the best of our knowledge, we set forth to optimize the enhanced, consistent and sufficient expression of unmodified human CYP1B1 in bacterial expression system for functional studies. We adopted a simple yet efficient methodology/protocol which can be executed in any laboratory without the requirement of a fermentor. Though we report the expression of CYP1B1 in this study but the protocol can, in principle, be applied to heterologous expression of any eukaryotic membrane protein.

## Material and Methods

### Chemicals

The plasmid pCWori(+) without the CYP1B1 construct and pCWori+CYP1B1 (modified with bovine N-17 N terminal) was a generous gift from Prof. Guengerich [30]. Primers were ordered from Sigma. Restriction enzymes were purchased from New England Biolabs. Anti-CYP1B1 monoclonal antibodies for the western blotting were purchased from Abcam (Cat # ab185954). LB agar, LB broth, tryptone, peptone and yeast extract were purchased from Difco BD Biosciences. Sequencing was outsourced from CromDx Solutions Private Limited. Glygerol and all other reagents were purchased from Sigma Life Sciences, Sigma-Aldrich and SD Fine-Chem Ltd.

***Vectors***: The expression of CYP1B1 was checked in three different bacterial expression vectors viz. pET28a(+), pCWori(+) and pTrcHis. Wild type CYP1B1 cDNA was cloned in all the three plasmid vectors and all the clones were subjected to expression protocols in order to evaluate the expression efficiency of each vector. ***Culture media***: Four different formulations of culture media were adopted to identify the one yielding best expression. These were LB broth, peptone based terrific broth (TB), tryptone based TB and a 1∶1 preparation of peptone/tryptone mixture in TB. The TB was prepared by dissolving 12 g peptone/tryptone (or 6 g each of peptone and tryptone in case of peptone/tryptone TB), 24 g yeast extract and 4 ml glycerol in double distilled water to make a final solution of 900 ml. The resultant solution was then autoclaved and allowed to cool. After that 100 ml of freshly prepared 0.17 M KH_2_PO_4_+0.72 M K_2_HPO_4_ solution was added to make the final volume 1000 ml. ***Trace element solution***: Assessment of the role of trace element solution (TES) in heterologous CYP1B1 expression was also carried out. TES was prepared as follows: Citric acid∶H_2_O-(5 g), ZnSO_4_∶7H_2_O-(5 g), Fe(NH_4_)_2_(SO_4_)_2_∶6H_2_O-(1 g), CuSO_4_∶5H_2_O-(0.25 g), MnSO_4_∶H_2_O-(0.05 g), H_3_BO_3_, anhydrous-(0.05 g) and Na_2_MoO_4_∶2H_2_O-(0.05 g) was dissolved in double distilled water to form the final solution of 100 ml. TES was always prepared fresh in order to avoid the use of preservatives (like chloroform) that may thwart protein expression. The composition of TES and TB is given in Table 1. ***E. coli strains***: CYP1B1 expression was examined in six different *E. coli* strains viz. DH5α, JM109, C100, DE3, Codon Plus, Pril.

**Table 1:** Composition of the trace element solution and the various combinations of terrific broth used in the experimental setup.

**Trace Element Solution (100 ml)**		
S. No.	Reagent	Quantity
1	FeCl3∶6H2O	2.7 g
2	ZnCl2∶4H2O	0.2 g
3	CoCl2∶6H2O	0.2 g
4	NaMoO4∶2H2O	0.2 g
5	CaCl2∶2H2O	0.1 g
6	CuCl2	0.1 g
7	H3BO4	0.05 g
8	HCl (Conc)	10 ml
9	Double distilled water	To make the final volume of 100 ml
**Terrific Broth (1000 ml)**		
1	Tryptone/peptone/tryptone∶peptone	12 g/12 g/6 g∶6 g
2	Yeast extract	24 g
3	Glycerol	4 ml
4	0.17 M KH2PO4+0.72 M K2HPO4	100 ml
5	Double distilled water	To make the final volume of 1000 ml

### Time Gradients

Influence of time on expression of CYP1B1 was monitored for different time points after induction. The time points at which the harvest was evaluated were 12 hours, 20 hours, 24 hours and 30 hours. ***IPTG Gradient***: The heterologous protein expression was checked both without IPTG and with an IPTG gradient. The IPTG gradient tested was 0 mM, 0.2 mM, 0.4 mM, 0.6 mM and 0.8 mM. ***RPM***: Though rpm is not generally considered to be an important modifiable factor in membrane protein expression but we evaluated its role also. We checked the expression at 150, 170, 200 and 250 rpm using New Brunswick Scientific Innova incubater-shaker. ***Temperature***: Four different temperatures viz. 18°C, 25°C, 30°C, and 37°C were employed to test out for the temperature at which CYP1B1 is expressed the best.

### Conditioning agents

Since CYP1B1 is a heme containing protein, we also assessed the impact of various conditioning agents (including heme precursors) in enhancing the expression. We analyzed the expression with and without δ-amino levulenic acid (ALA) and thiamine. We also employed the gradient of these conditioning agents at 0.2 mM, 0.4 mM, 0.6 mM, 0.8 mM and 1.0 mM for ALA and 0.5 mM, 1.0 mM and 1.5 mM for thiamine.

### Double colony selection

As observed by Sivashanmugam et al., (2009) [31] “double colony selection” is one of the most important factors for significantly higher amounts of heterologous expression of recombinant proteins. We adopted the protocol they have used for combating the problem of low protein yields in glycerol stocks. Briefly, a single freshly transformed colony grown on LB agar was inoculated in LB medium and allowed to grow in shaker incubator at 37°C and 200 rpm to an OD of 0.6. A small amount of this culture was spread on an agar plate. Then colonies from the plate were selected and again inoculated in LB medium and allowed to grow after IPTG induction. After that 350 µl of the culture medium containing the cells was centrifuged at 3000 g for 12 minutes and the pellet was prepared for SDS-PAGE. The protein expression was observed in SDS-PAGE followed by commassie brilliant blue staining. Colonies expressing higher levels of protein were selected for the next step (which was just a repetition of the above mentioned procedure). Colonies showing highest protein expression were then selected for subsequent experiments.

### Cloning of CYP1B1

The CYP1B1 cDNA was prepared from mRNA isolated from peripheral blood leukocytes. For this a 3 ml blood sample was drawn from a healthy individual by venipuncture (by a trained phlebotomist) after proper/written informed consent. The study was approved by the Institutional Ethics Committee, All India Institute of Medical Sciences, New Delhi vide letter No. IESC/T-025/2011. RNA was isolated with TRIzol Plus RNA Purification Kit (Life Technologies) according to the manufacturer's instructions. After first strand cDNA synthesis, the cDNA was subjected to amplification. Using specific primers, *NdeI* (ca^↓^tatg) and *XbaI* (t^↓^ctaga) cloning sites were introduced as reported by Jansson et al. (2000) [22]. The primer sequence is Forward seq: 5′-gcg c***ca tat g***gg cac cag cct cag ccc gaa cga ccc-3′ and Reverse seq: 5′-gcg c***tc tag a***tt att ggc aag ttt cct tgg ctt gta-3′. Polymerase chain reaction was carried out in a total 50 µl reaction volume and subjected to thermocycling in a BioRad C1000 thermal cycler. The reaction conditions were 94°C for 90 seconds (denaturation), 54°C for 42 seconds (annealing) and 66°C for 70 seconds (extension). This cycle was repeated 36 times with a final extension of 94°C for 60 seconds. The amplification product was subjected to electrophoresis on 1% agarose gel containing ethidium bromide and visualized in the Fujifilm Las 4000 Gel Documentation Unit. The PCR product was purified and cloned separately into the three vectors described in the relevant section. After cloning, each plasmid was separately transformed in already mentioned strains of *E. coli* and plated on ampicillin (pCWori(+) and pTrcHis) or kanamycin (pET28a+) containing agar plates and kept at 37°C for 12 hours. After colonies appeared, a single colony from each plate was picked and separately inoculated into 100 µg/ml ampicillin or 50 µg/ml kanamycin (depending on vector) containing LB broth and allowed to grow for 14 hours. Miniprep plasmid isolation was done by Geneaid High Speed Plasmid Minikit (Cat# PD100) according to manufacturer's instructions. The resultant isolated plasmids were subjected to restriction digestion by *NdeI* and *XbaI*. The digestion mixture was then electrophoresed on 1% agarose gel containing ethidium bromide to check the correct sized pop-out. Positive clones were subsequently sent for sequencing for further confirmation.

### N-Terminal modification

Though N-terminal modification has been reported to improve the yield of recombinant cytochrome P450s and other membrane proteins but we did not employ any N-terminal modification. This is because we wanted to get a higher and consistent yield of the native (unmodified) CYP1B1 protein suitable for scientifically valid functional investigations and viable biophysical studies.

### SDS-PAGE and Western Blot

For SDS-PAGE a 3 ml aliquot of the bacterial cell suspension was centrifuged at 3,000 g for 12 minutes. The supernatant was discarded and the pellet was resuspended in 1 ml solution containing 30 mM Tris (pH = 8.0), 300 mM NaCl, 25% sucrose, 1% triton-X and 1 mM PMSF. The resultant suspension was kept at room temperature for 20 minutes and then subjected to sonication. Sonication pulses were given in repeated cycles till the suspension became clear (taking care that the suspension does not heat up). The suspension was then centrifuged at 16,000 g for 15 minutes. The supernatant was treated with loading dye (containing 50 mM Tris, 1% β-mercaptoethanol, 10% glycerol, 2% SDS and 0.02% bromophenol blue). Protein electrophoresis was then carried out on an SDS-PAGE (10% resolving and 5% stacking gel). The gels were then either stained overnight with commassie brilliant blue and developed with repeated washing using a destaining solution (40% glacial acetic acid, 50% methanol and 10% water) or were subjected to western blotting.

For western blotting the electrophoresed proteins in the gel were transferred to a nitrocellulose membrane and then developed according to the method described by Towbin et al. (1979) [32] with slight variations. Western blotting was carried out using anti-CYP1B1 monoclonal antibodies and anti-HRP conjugated 2° antibodies.

### Protein estimation

Total protein was estimated by measuring the optical density at 280 nm by Nanodrop spectrophotometer (ND-1000). Cytochrome P450 measurement was carried out as per the protocol reported by Jansson et al. (2000) [22] and spectral measurement were taken as reported by Schenkman and Jansson (1998) [33] using Jasco V-660 spectrophotometer. The CYP1B1 recombinant protein was estimated by densitometric analysis using Fujifilm Las 4000 Gel Documentation Unit utilizing Image-Quant software.

## Results and Discussions

**Figure 1:**
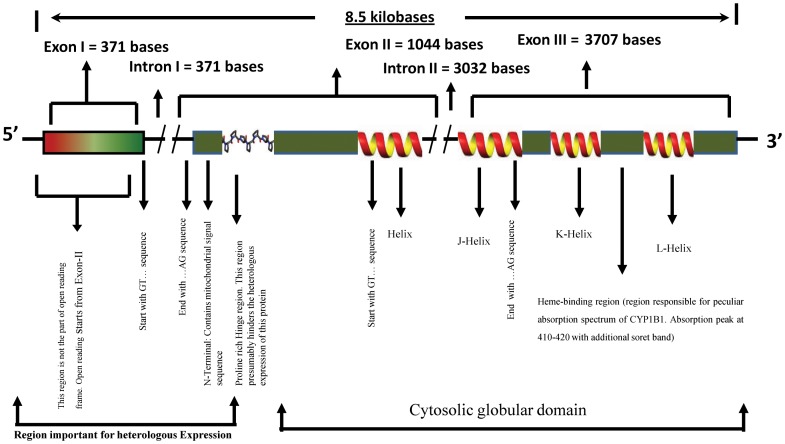
Schematic Diagram of CYP1B1 gene. The CYP1B1 gene has 2 introns and 3 exons. The open reading frame starts from Exon II and ends within Exon III. The gene codes for a 543 aminoacid protein with a membrane spanning domain at the N-terminal followed by a proline rich hinge region which in turn is followed by the cytosolic globular domain. The chromosomal location of the gene is 2p21–22.

Eukaryotic membrane proteins are difficult to express in heterologous hosts. This is especially true for transmembrane proteins [34]. CYP1B1 expression is particularly difficult in this regard and, therefore, several studies have been carried out using N-terminal modified protein [22], [23], [25]–[29]. Hence, it is necessary to develop a protocol that expresses the full length protein in sufficient quantities for functional studies. Additionally, proline is thought to be poorly translated in *E. coli* independent of its position in the translation product and regardless of the codon bias [35]. Interestingly, CYP1B1 has a proline rich hinge region towards the N-terminal region near the transmembrane domain to allow flexibility (Fig. 1). This phenomenon presumably further contributes towards the difficulties encountered in heterologous CYP1B1 expression. Investigators reporting CYP1B1 expression have circumvented these hindrances by N-terminal modifications [22], [23]. This is because N-terminal modifications have been reported to enhance heterologous expression of eukaryotic membrane proteins. A reasonable explanation for this observation is that eukaryotic mechanisms for translation initiation are extensively dissimilar to that of prokaryotes [36]. Hence, N-terminal sequences seem to be an important decisive factor in heterologous protein expression and that is why investigators pay attention to its modification. Literature suggests that initial 15–25 codons of the open reading frame deserve extensive deliberation for optimization of expression [37]–[45]. Such a method yields good expression but N-terminal specific functions of the protein are lost. As a representative instance, it will be conceptually warranted to mention here that the founder mutation G61E (an N-terminal mutation contiguous to the N-terminal proline rich hinge area) is deleterious to protein function and results in disease phenotype [46]. It is the proline-proline-glycine-proline motif that is presumably involved in joining of the N-terminal region of CYP1B1 with the globular domain for proper functioning of the CYP1B1 protein [17], [47]–[49]. Such effects may change a number of characteristics of the expressed protein involving cell signaling, enzymatic activities, functional characteristics, protein stability, charge, structure and many other functions. It is, therefore, always desirable to express the protein of interest with its native aminoacid sequence. This becomes particularly important in mutational studies and more so when the mutations are in the N-terminal region. Inspired by this reasoning, we undertook the important task of expressing native (unmodified) CYP1B1 in sufficient quantities in *E. coli*.

**Figure 2:**
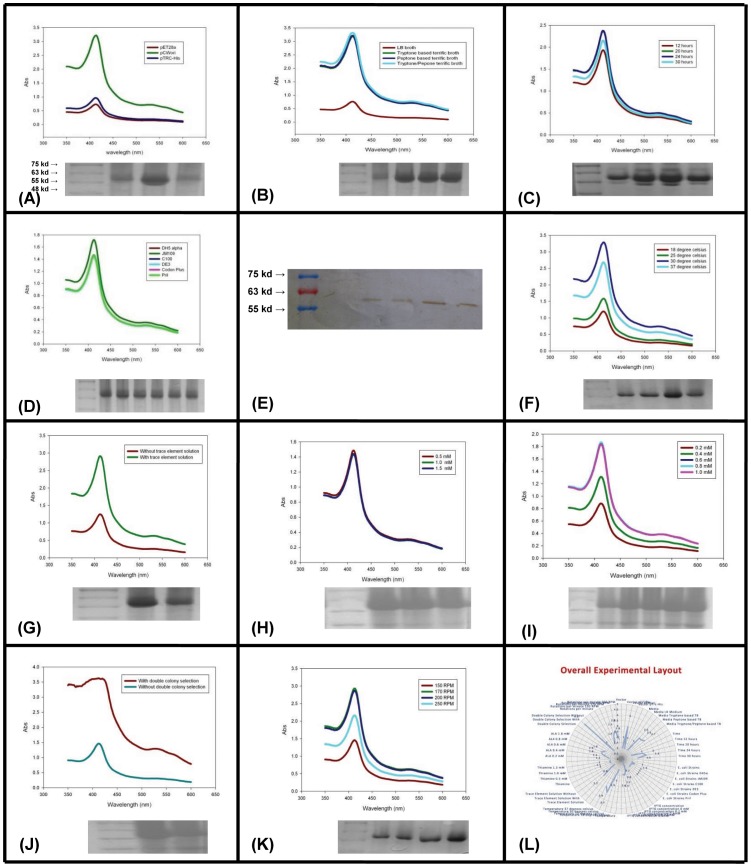
PAGE/Western blot pictures of CYP1B1 expression for various variables with absorption spectrum adjoining. Each unit shows absorption spectrum of the expressed protein at 350 to 600 nm. Each absorption spectrum shows a peak somewhere around 410–420 nm which is characteristic of the heme present in the CYP1B1 protein. The colours in each graph has a defined sequence which correlates with the SDS-PAGE picture shown just underneath each absorption curve. In every graph, the dark red colour corresponds to the second lane of the gel picture underneath (lane next to marker which is the first lane starting from the left). The dark green corresponds to the third lane, dark blue to the fourth, cyan colour to the fifth, sixth is pink and seventh is the fluorescent green. (**A**) CYP1B1 expression with three different vectors viz. pET28a+, pCWori(+) and pTRC-His. (**B**) CYP1B1 expression with different media viz. LB-broth, tryptone based TB, peptone based TB and TB made with 1∶1 mixture of tryptone and peptone. (**C**) CYP1B1 expression at different time points viz. 12 h, 20 h, 24 h and 30 h (**D**) CYP1B1 expression with six different strains of *E. coli* viz. DH5α, JM109, C100, DE3, Codon Plus and Pril. (**E**)Western blot showing the increase in protein expression with increasing IPTG concentration. Starting from left is marker folowed by expression without IPTG induction and then folowed by 0.2 mM, 0.4 mM, 0.6 mM and 0.8 mM IPTG concentrations. (**F**) Expression pattern of CYP1B1 at four different temperatures viz. 18°C, 25°C, 30°C and 37°C. (**G**) CYP1B1 expression without and with TES. (**H**) CYP1B1 expression pattern with different concentrations of thiamine viz. 0.5 mM, 1.0 mM and 1.5 mM. (**I**) Expression pattern of CYP1B1 at different δ-ALA concentrations viz. 0.2 mM, 0.4 mM, 0.6 mM, 0.8 mM and 1.0 mM. (**J**) CYP1B1 expression with and without double colony selection. (**K**) Expression pattern of CYP1B1 at four different rpm levels viz. 150, 170, 200, 250. (**L**) Overall experiment layout with all the variables and results obtained.

It is well known that prokaryotic expression plasmids contain customized and optimal genetic elements that are important in favorable recombinant protein production at both transcriptional as and translational levels. We made use of different vectors to identify the plasmid that best expresses our protein of interest. CYP1B1 was expressed in pCWori (+) as is evident from Fig. 2(A). Choice of the appropriate expression vector is an important decisive factor for heterologous protein expression and an investigator may need to repeat experiments with many vectors in order arrive at the precise pick. In our case pCWori(+) happened to be the vector of choice. Our results with this vector are in agreement with previously published studies [22]. pCWori (+) is a *tac* promoter containing expression vector that has been specially used for expression of cytochrome P450s without any fusion tag.

Culture medium should be meticulously formulated as it may affect the cell growth and metabolism. Therefore, culture medium composition may have significant effects in the protein yield. To add to this, translational efficiency of various mRNAs is influenced by culture media composition and changes in temperature [50]. For this reason we conducted our experiments with different media compositions and for different periods of time. We observed that our protein of interest does not express in LB-media and requires TB for expression. It is clear from Fig. 2(B) that a 1∶1 mixture of peptone/tryptone based TB (with 1% glucose) showed best expression. This may be because certain proteins require additional supplements for expression. Additional supplementation may be needed for bacterial growth to sustain the recombinant protein expression. Bacterial growth and excess recombinant protein production leads to substrate depletion, pH changes and accumulation of inhibitory factors [25]. As a consequence, use of TB-media becomes essential in some cases. Additionally, CYP1B1 takes longer time (post induction) to express. Hence the need for TB media is further justified. This is because long time of expression often leads to depletion of substrates, accumulation of metabolic byproducts and, in consequence, plasmid instability. Specially formulated culture media optimized to the production of a particular protein often becomes a necessity and media modification often shows better yields [51], [52]. Eukaryotic membrane proteins usually need longer time to express in *E. coli* which often results in decreased plasmid stability. In order to circumvent this problem, we buffered the culture media by adding 0.17 M KH_2_PO_4_+0.72 M K_2_HPO_4_ solution to the TB. Maximum yield of CYP1B1 protein was observed at 24 hours of induction (Fig. 2(C)) while it decreased beyond 24 hours probably due to exhaustion of buffering capacity of the media and/or due to accumulation of the overexpressed protein and consequently its increased degradation. Extended time of expression and bacterial growth may also lead to the depletion of the nutrients and supplements essential for recombinant protein production. Furthermore, accumulation of certain toxins may also contribute to this phenomenon. On the other hand, shorter induction time did not produce enough protein. We got the highest yield of CYP1B1 at 24 hours indicating that an investigator needs to check as many time points as possible so as to arrive at the appropriate time point at which his/her protein is expressed in sufficient quantities.

Fig. 2(D) shows that all the *E. coli* strains expressed the same quantities of protein except for JM109 strain which yielded a slightly higher amount. Similar expression amount presumably is a result of similarities in the transcription and translation mechanisms in these bacterial strains. No enhancement in expression amounts in Codon plus indicates that codon bias may not be a significant problem for CYP1B1 expression. No change in recombinant protein expression in DE3 signifies that mRNA translation (not transcription) is the limiting step in CYP1B1 expression. DE3 contains bacteriophage T7 RNA polymerase (T7 RNAP) which has higher transcription rate than *E. coli* RNAP because T7 RNAP transcription is controlled by *lac*UV5 promoter which is a mutant (and more powerful) of wild-type *lac*-promoter [53]. As Schlegel et al., [54] suggest this should increase the protein expression but may not always be true. For protein of our interest, this option did not work. However, that does not mean DE3 should not be used for overexpression of other membrane proteins. Interestingly, JM109 did produce slightly higher amounts apparently due to enhanced plasmid stability. JM109 possesses hsdR17 genotype thereby preventing the degradation of heterologous DNA (expression plasmid in this case) by endogenous endonuclease.

In our study, we observed that higher IPTG concentration enhanced expression but was ineffective beyond 0.6 mM. The western blot shown in Fig. 2(E) reveals the effect of IPTG on CYP1B1 expression. An immediate interpretation of this observation is that increasing IPTG concentration leads to corresponding increase in transcriptional activity till a saturation stage is reached beyond which no further increase is possible. With regards to temperature, heterologous expression is generally carried at 37°C but in certain cases lowering of temperature becomes beneficial by increasing protein stability and enhancing correct folding patterns [25]. Lowering temperature also leads to a decline in recombinant protein degradation rate [51], [55], . On the other hand, lowering the temperature also reduces bacterial metabolism and consequently protein expression; this includes recombinant expression also. Therefore, a balance needs to be stricken at the optimum temperature a particular recombinant protein best expresses at. In our studies CYP1B1 was best expressed at 30°C with higher protein degradation at 37°C and reduced expression at lower temperatures (Fig. 2(F).

We observed very low protein yields without the addition of the TES irrespective of presence of all other factors. Fig. 2(G) reveals the difference between CYP1B1 expression with and without the use of TES. In addition to the carbon source, a good media contains other elements which are depleted as the bacteria increase in number. Therefore, the addition of trace elements such as sodium, potassium, magnesium, ammonia, sulphates, iron (important for heme synthesis) and other nitrogenous complexes must be supplied. There are different compositions of TES reported in literature; the one we used is given in Table 1. In our study, thiamine did not show any enhancement in the expression neither did it hamper the same (Fig. 2(H)). This may be due to the fact that *E. coli* produce their own thiamine and any additional supply of thiamine does not confer any advantage for CYP1B1 expression. However, we speculate that thiamine should be evaluated as an additive to culture media used to express heme containing proteins. Interestingly, we observed that addition of ALA to the induction medium enhances CYP1B1 expression (best at 0.8 mM). It is surprising to see that ALA enhances protein expression in spite of the fact that *E. coli* synthesize sufficient amounts of ALA to support excessive cytochrome P450 production [29]. Fig. 2(I) shows the effect of ALA on CYP1B1 expression. Though we could not investigate the reasons for this observation but we speculate that addition of ALA may possibly relieve the transformed *E. coli* from the metabolic burden of energetically expensive synthesis of ALA.

Double colony selection enhanced the production of CYP1B1 approximately five times. It is an extremely important step that has not been previously utilized for CYP1B1 expression and expression of a majority of other membrane proteins. This method improves the yield without much difficulty and need of any additional chemicals and skills. We foresee this method being employed in all cases where the heterologous protein yield is not sufficient. It is a reliable and easy method with consistently promising results as shown in Fig. 2(J). RPM does not generally influence protein expression but it happened to affect the amount of yield in our case. We observed the best yield at 170 rpm with least yield at 150 rpm and second least at 250 rpm. A probable explanation of this observation can be that lower rpm may lead to sedimentation of bacteria and too high RPM might lead to increased protein degradation and cell death due to mechanical shear; Fig 2(K). Schematic trend of the effect of various factors are summed up in **Fig S1** which also gives a panoramic view of the experimental layout.

## Conclusion

Heterologous expression of unmodified CYP1B1 in *E. coli* is difficult and, therefore, hampers research to evaluate important aspects of many cancers, pathomechanistic studies of PCG and understanding of various metabolic pathways. This in turn leads to impediments in development of therapeutic regimens and design of effective drugs needed to treat various disorders. Expression and purification of sufficient amount of CYP1B1 is crucial for many areas of biomedical research. Here we report the development of a protocol which reveals that culture in TB media (preferably 1∶1 peptone + tryptone), TES, ALA (0.8 mM) at 30°C and 170 rpm done in combination with double colony selection yields high amounts of CYP1B1 with consistency and experimental repeatability. The complete experimental overview is given in Fig. 2(L) and experimental setup with various variables and results is shown in Table 2. This protocol, though used primarily for CYP1B1, is a useful and user friendly method to achieve good recombinant protein expression (especially for membrane protein) consistently in *E. coli*.

**Table 2:** Panoramic view of the experiments carried out with the numerical values of the variables and the results obtained.

Variable	Gradient Experiment	Variable with Best Expression
**Vector**	[pET28a+]: [pCWori(+)]: [pTrc-His]	**pCWori(+)**
**Medium**	[LB Broth]: [Tryptone TB]: [Peptone TB]: [Peptone + Tryptone TB]	**Peptone + Tryptone TB**
**Time**	[12 hours]: [20 hours]: [24 hours]: [30 hours]	**24 hours**
***E. coli*** ** Strains**	[DH5α]: [JM109]: [C100]: [DE3]: [Codon Plus]: [Pril]	**JM109**
**IPTG**	[0 mM]: [0.2 mM]: [0.4 mM]: [0.6 mM]: [0.8 mM]	**0.6 mM**
**Temperature**	[18°C]: [25°C]: [30°C]: [37°C]	**30°C**
**TES**	[Without]: [With]	**With**
**Thiamine**	[0.5 mM]: [1.0 mM]: [1.5 mM]	**No difference**
**ALA**	[0.2 mM]: [0.4 mM]: [0.6 mM]: [0.8 mM]: [1.0 mM]	**0.8 mM**
**Double Colony Selection**	[With]: [Without]	**With**
**RPM**	[150]: [170]: [200]: [250]	**170**

The best fit in each variable is also shown in the rightmost column.

## Supporting Information

Figure S1 **Panoramic view of the experiments, methods and resultant trends in CYP1B1 expression due to variations in various relevant factors.**
(PPT)
