# Supplementary figures and images for: A Novel Methodology for Enhanced and Consistent Heterologous Expression of Unmodified Human Cytochrome P450 1B1 (CYP1B1)
Source: PLoS One. 2014 Oct 16;9(10):e110473. doi: 10.1371/journal.pone.0110473 (PMC4199734; doi:10.1371/journal.pone.0110473)

## Slide 1
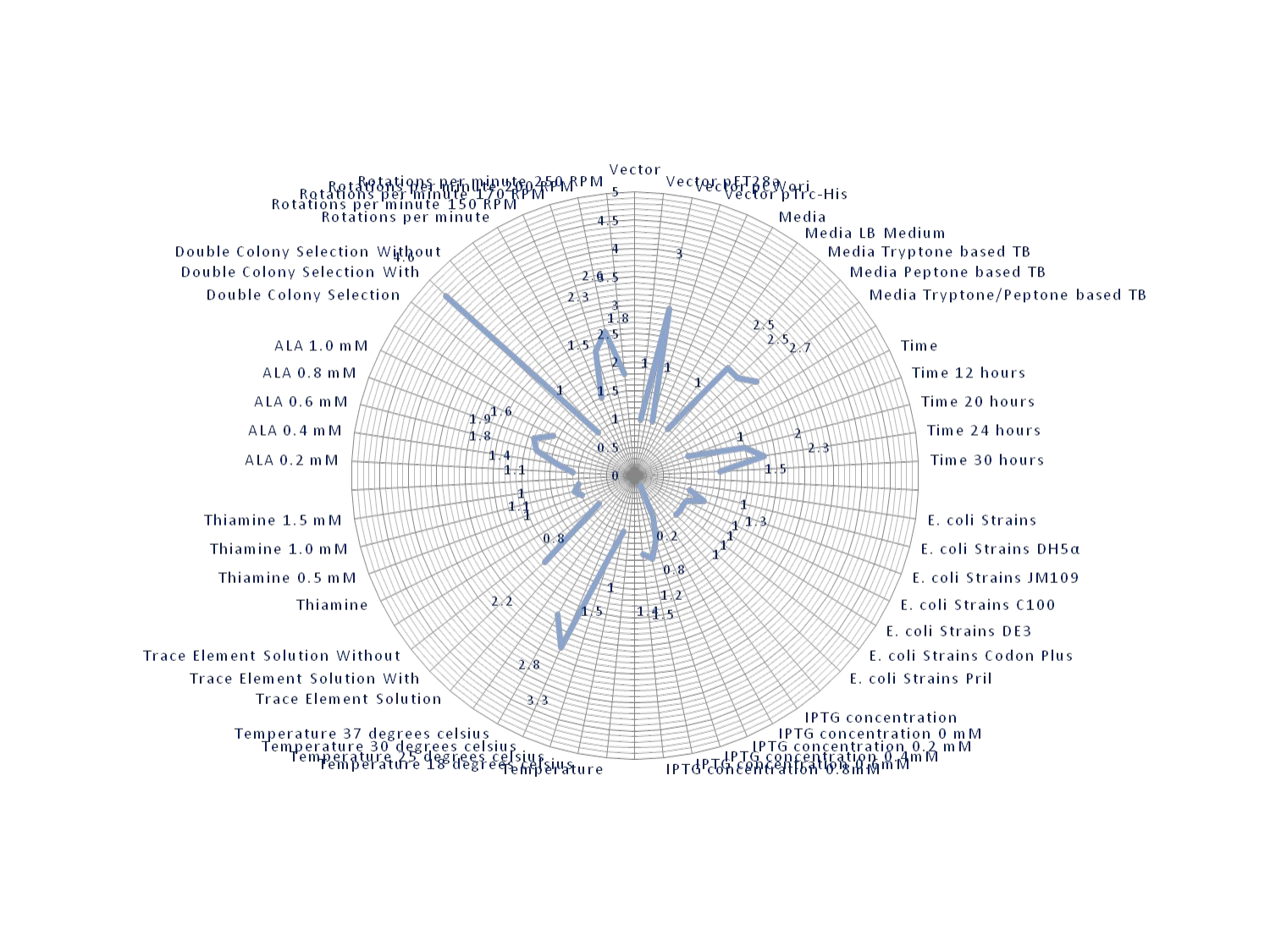

Supplement: Figure S1 — Panoramic view of the experiments, methods and resultant trends in CYP1B1 expression due to variations in various relevant factors. (PPT) [file pone.0110473.s001.ppt]
